# Hydration and Barrier Potential of Cosmetic Matrices with Bee Products

**DOI:** 10.3390/molecules25112510

**Published:** 2020-05-28

**Authors:** Jana Pavlačková, Pavlína Egner, Roman Slavík, Pavel Mokrejš, Robert Gál

**Affiliations:** 1 Department of Lipids, Detergents and Cosmetics Technology, Faculty of Technology, Tomas Bata University in Zlín, Vavrečkova 275, 76001 Zlín, Czech Republic; pavlackova@utb.cz (J.P.); egner@utb.cz (P.E.); 2 Prodejní místa, Alveare, Ltd., Štěpnická 1137, 68606 Uherské Hradiště, Czech Republic; slavik@post.cz; 3 Department of Polymer Engineering, Faculty of Technology, Tomas Bata University in Zlín, Vavrečkova 275, 76001 Zlín, Czech Republic; 4 Department of Food Technology, Faculty of Technology, Tomas Bata University in Zlín, Vavrečkova 275, 76001 Zlín, Czech Republic; gal@utb.cz

**Keywords:** bee products, bioactive molecules, cosmetics, emulsion, functional matrices, honey, hydration, organoleptic properties, transepidermal water loss

## Abstract

Honey, honey extracts, and bee products belong to traditionally used bioactive molecules in many areas. The aim of the study was primarily to evaluate the effect of cosmetic matrices containing honey and bee products on the skin. The study is complemented by a questionnaire survey on the knowledge and awareness of the effects and potential uses of bee products. The effect of bee molecules at various concentrations was observed by applying 12 formulations to the skin of the volar side of the forearm by non-invasive bioengineering methods on a set of 24 volunteers for 48 h. Very good moisturizing properties have been found in matrices with the glycerin extract of honey. Matrices containing forest honey had better moisturizing effects than those containing flower honey. Barrier properties were enhanced by gradual absorption, especially in formulations with both glycerin and aqueous honey extract. The observed organoleptic properties of the matrices assessed by sensory analysis through 12 evaluators did not show statistically significant differences except for color and spreadability. There are differences in the ability to hydrate the skin, reduce the loss of epidermal water, and affect the pH of the skin surface, including the organoleptic properties between honey and bee product matrices according to their type and concentration.

## 1. Introduction

Bees are important to humans not only by pollinating many species of plants, thereby helping to multiply them, but also by creating unique products containing a range of bioactive molecules that have been used for centuries in human nutrition, folk medicine, pharmacy, and cosmetics. Bee products may be of both vegetal and animal origin. The products of vegetal origin include those molecules that bees collect in the wild and bring to the hive, where they process them for their needs. These include propolis—the compound from the flower and leaf buds of alder, birch and other trees, pollen—the floral pollen of flowering plants and honey—the nectar of flowering plants, or honeydew produced by the *Homoptera insecta*. Bee products of animal origin include substances that the bee itself secrete in its own body. It is bee venom—secretion of the venom gland, royal jelly —secretion of the pharyngeal and mandible glands of worker bees, and beeswax—the secretion of wax-forming glands of worker bees [1,2]. The composition, color, aroma, and biological activity of bee products depend on the location, time, and source of the plant from which they are obtained.

Among the raw materials for cosmetic matrices, honey is the most important. “Honey” or “Mel” is included in the International Nomenclature of Cosmetic Ingredients (INCI) as an moisturizing/humectant/emollient product [3]. Honey is useful in products of skin care, and its regular application contributes to the skin juvenility and the reduction of wrinkle formation [4]. Honey is used in variable proportions according to the type of cosmetic. Generally, a lower concentration (0.5–5%) is used for foams, creams and emulsions, while a higher concentration (10–15%) is used for anhydrous ointments. Honey is most commonly used in the range of 1% to 10% in products such as lip ointments, cleansing milk, hydrating creams or gels, after-sun products, tonic lotions, shampoos, and conditioners. Higher concentrations (up to 70%) can be used for combinations of honey with oils, gelling agents, and emulsifiers or in face masks [5,6]. Honey is also used as an alternative to traditional emulsifiers in body lotions for bathing and shampooing, where they make up 50% to 50% surfactants [7].

Beeswax is used to modify the texture of cosmetic products: 1–3% for creams and ointments, balms, and lotions, 6–12% for mascara, and 6–20 % for eye shadow. It is included in deodorants (up to 35%), depilatory preparations (up to 50%), hair cosmetics (1–10%), lipsticks (10–15%), and other products. Its presence also improves the stability of the formulations. Another bee product used primarily for its antimicrobial effects is pollen, which is added to dry shampoos, creams, and tonics [8]. For its anti-aging effect, a royal jelly is widely used. Royal jelly extract increases the natural moisturizing factor. The addition of 0.05% to 1.0% stimulates and nourishes the epidermis; it is used e.g., in face lotions, body milk, hair cosmetics, and soaps [9]. Propolis has regenerative, antioxidant, anti-inflammatory, antiseptic, antifungal, bacteriostatic, astringent, antispasmodic, anesthetic, anticancer and photoprotective effects [10,11]. It is added in a concentration of 1% to 2% to aftershave, after-bath and oral care products, shampoos, deodorants, soaps, and creams [12].

The development of bee products for dermal applications may take different directions in the future. Burlando and Cornara [7] see one way in ethnopharmaceuticals surveys focused on significant biological properties in the extraordinary variety of mono- and polyfloral honeys. Another possibility is to carry out chemical and biological research focused on the chemical composition of honey and its pharmacological efficacy, thus opening the way to new medical procedures supporting human health [13].

The use of honey, honey extracts, and bee products as bioactive molecules in many cosmetic products (especially body and hair cosmetics) has been known for some time. A variety of publications describe the diverse effect of these formulations; however, none of them quantitates the moisturizing and barrier effect containing various bee products. The aim of the present paper is to assess (1) the moisturizing and barrier properties of emulsion matrices with the addition of honey and bee products on the skin; (2) perform sensory evaluation of the emulsion matrices with the addition of honey and bee products; and ascertain (3) by means of a questionnaire survey the effects of cosmetic matrices with the addition of honey and bee products, the reasons and frequency of use of honey cosmetics. The specific hypotheses tested are as follows. (1) Higher skin hydration may be expected for emulsion matrices with a higher content of honey or bee products. (2) Emulsion matrices with a higher honey and wax content may be expected to have a difference in their spreadability. (3) Honey and/or bee products are expected to be popular with female respondents.

## 2. Results and Discussion

### 2.1. Biophysical Characteristics

**Figure 1:**
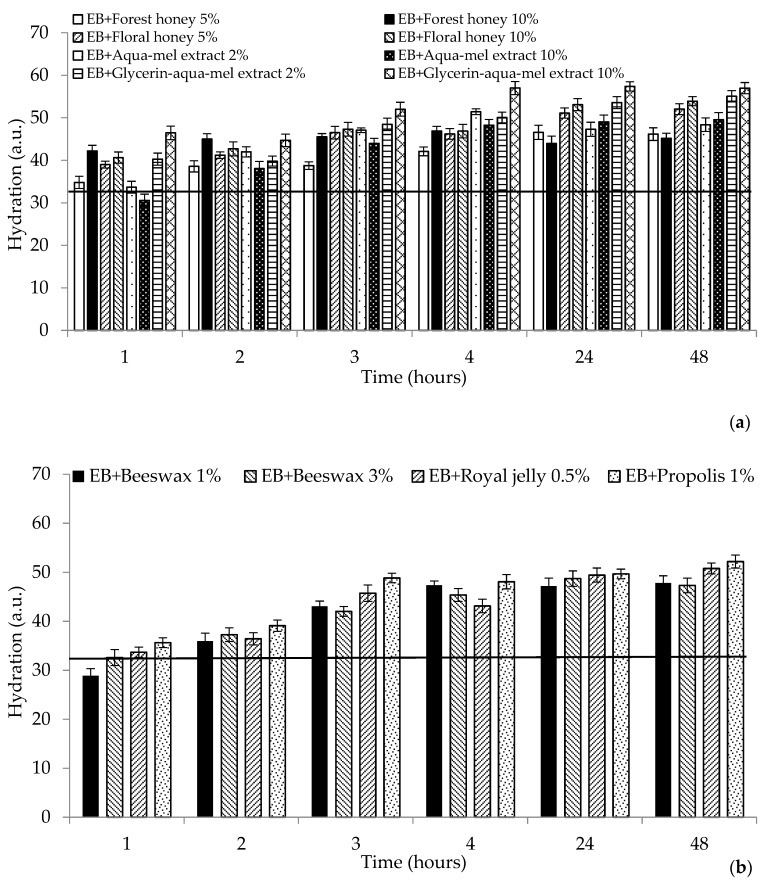
Hydration effect of tested cosmetic matrices after sodium lauryl sulfate (SLS) pretreatment (continuous horizontal line) over the studied period: (**a**) honey and honey extracts (**b**), other bee products. EB: emulsion base.

**Figure 2:**
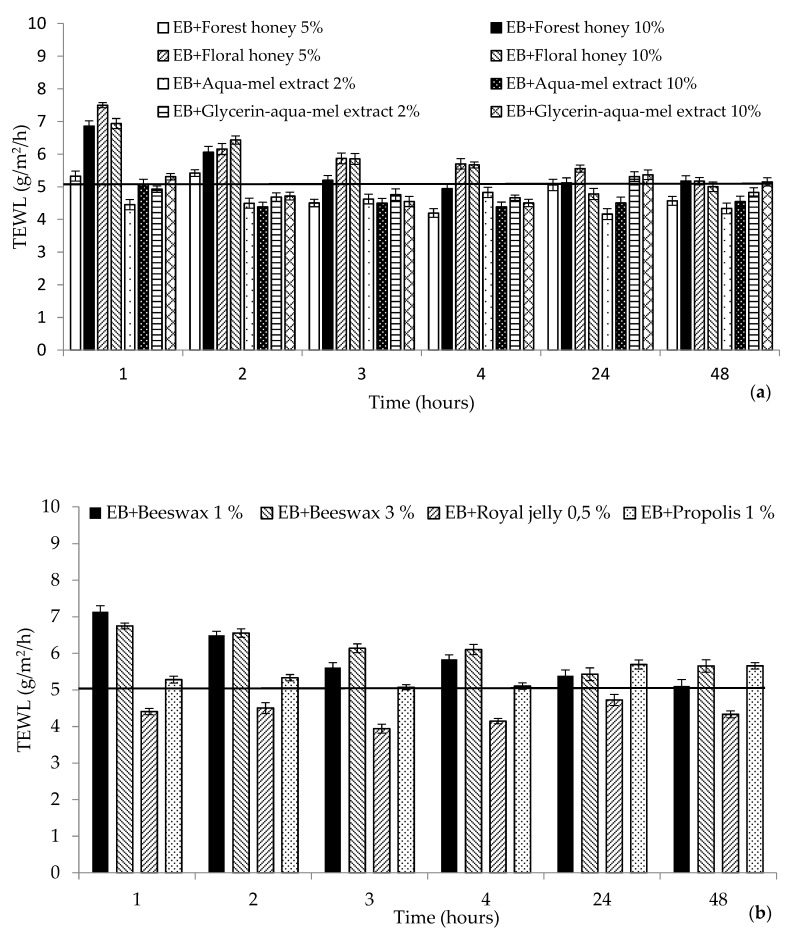
Transepidermal water loss (TEWL) after SLS pretreatment (continuous horizontal line) and application of the tested cosmetic matrices over the studied period: (**a**) honey and honey extracts (**b**), other bee products. EB: emulsion base.

**Figure 3:**
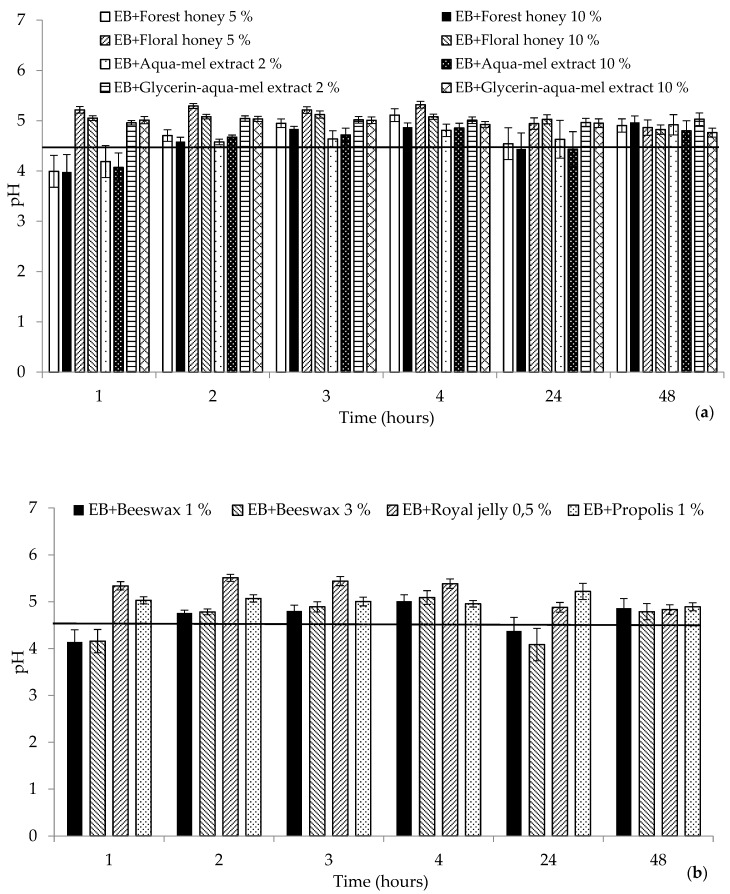
Values for pH after SLS pretreatment (continuous horizontal line) and application of the tested cosmetic matrices over the studied period: (**a**) honey and honey extracts (**b**), other bee products. EB: emulsion base.

The measurement of hydration, barrier, and pH effects on the skin due to the effect of prepared cosmetic matrices were preceded by a 0.5% sodium lauryl sulfate (SLS) skin pretreatment, the so-called washing test, which simulated the use of cosmetics during personal hygiene, such as showers or washes, and another purpose was to eliminate differences in skin properties at the site of intended application. The skin pretreatment is presented in the Figure 1, Figure 2 and Figure 3, characterizing the measured parameters by a continuous horizontal line corresponding to the average value of the monitored parameter measured before the emulsion application. This method of pretreating the skin with SLS solution in various concentrations is referred to in a number of works devoted to testing various preparations [14,15,16].

The hydrating ability of emulsion matrices with honey, honey extracts, and other bee products is shown in Figure 1. Emulsion matrices with glycerin extract of honey have probably shown a synergistic hydrating effect of the ingredients contained in the extract; the final increase in hydration after degreasing the skin treated with both formulations was about 60%. Emulsions containing higher concentrations of bee products hydrated the skin more efficiently except for the aqueous extract formulation, where, on the contrary, the lower concentrations proved to be preferable after four hours of exposure. An interesting difference was observed during the treatment of forest and flower honey formulations, with flower honey showing a higher hydrating activity of approximately 15% after 24 h, which can be attributed to a slightly higher fructose and glucose content in flower honey (see Table 1). This is consistent with the results of Jiménez et al., which state that active hydration is influenced by the content of sugars, mainly fructose and glucose [17]. These sugars form hydrogen bridges with water and maintain the moisture of the skin horny layer. This creates a protective non-greasy film on the skin to help maintain water in the skin [18]. Burlando and Cornara [7] in their review extend this knowledge to the influence of other substances present such as amino acids and organic acids, which can supplement the natural moisturizing factors of the horny layer. It is known that the biological properties of a certain type of honey are determined by the nectar-producing plants; therefore, botanical resources are of great importance in cosmetics [19]. Honey with a higher content of better soluble fructose with respect to glucose is recommended as being more suitable for cosmetic products because of the lower risk of their crystalline form. There is a study [20] in which volunteers observe the hydrating effect of honey-containing emulsions from different bee species, where the *Apis mellifera* honey formulation proved to be the most hydrating active after two hours of application, which was followed by preparations containing the honey of *Melipona fasciculata* and the honey of *Tetragonula carbonaria* with the least hydrating effect. The skin treated with other bee product formulations also achieved a higher proportion of water in its corner layer. Hydrating effects of bee products are mentioned in publication [21]; the samples containing a higher amount of beeswax were absorbed into the skin more slowly than other tested formulations. The hydration of stratum corneum is crucial for the integrity and regulating the barrier properties.

The effect of emulsion matrices with honey, honey extracts, and other bee products on the skin barrier function is shown in Figure 2. The highest loss of epidermal water (TEWL) was monitored after treatment of the skin with an SLS solution that disrupted the skin barrier. This mechanism of increasing TEWL after treatment with diversely concentrated SLS solution is described by Tupker et al. [22]. Some authors [23] recommend replacing the SLS treatment with anionic phosphorus derivatives of alkyl polyglucsides that are more gentle to the skin barrier. Park and Eun [24] studied the concentration series of SLS solution, and the increase in TEWL was statistically significant relative to the higher concentration of the solution. The reduction of water loss from the epidermis proceeded gradually over the observed time intervals with the gradual absorption of emulsions. The higher increase in TEWL does not always correspond to higher concentrations of the studied active substances in cosmetic matrices. The reduction of dermal water loss was proved after the treatment of the skin with both glycerin and aqua mel extract during the 4 h of the experiment. In the study [25], the effects of aqua solution of glycerin in the concentration range 1–10% on the skin of women volunteers pretreated with 10% SLS solution under occlusion for 3 h was examined. Even at 2% concentration of glycerin, the water-holding capacity was enhanced. When evaluating matrices containing flower and forest honey, less water loss was observed after the treatment of the skin with a formulation with the addition of forest honey. In a randomized controlled trial by Zahmatkesh et al. [26], a mixture of olive oil, sesame oil, and honey was demonstrated to be a useful treatment for burns, by preventing infections, accelerating tissue repair, and facilitating debridement. Among other bee products proven to be suitable were emulsion formulations with wax and royal jelly, which have been shown to prevent the dehydration of the skin.

The last observed parameter affected by the matrices prepared was skin pH. Figure 3 shows a pH shift to the neutral area after application of the tested matrices, most notably for honey and beeswax formulations. Tested cosmetic matrices do not disturb the natural pH of the skin. The skin surface is naturally slightly acidic; the pH ranges from 4.0 to 5.5 depending on the location [27]. The skin pretreatment by degreasing represented a potential risk of removing dermal lipids and bacterial flora, to which Seweryn el al. [28] draw attention. From the pH values after the topical application of matrices containing honey and bee products, it can be concluded that no disruption of natural acid skin mantle was detected, which enables an indirect prediction of the influence of studied matrices on the skin. There was no irritating reaction (assessed visually) on the skin treated with the tested matrices, although honey-based cosmetics and cosmetics made of other bee products can function as sensitizers, as described in some publications [29,30]. To conclude, from a physiobiological approach, a casual dry skin may be treated with honey and other bee products matrices giving good efficacy.

### 2.2. Sensory Analysis

There were no statistically significant differences between the cosmetic matrices of the honey and bee products formulations assessed by the ranking test evaluating overall sample preference. Statistically significant differences at the 95% level of significance were found in the ranking test assessing the color of the matrices between samples AG, CF, CG, CE, CH, BG, BE, BH, DA, DF, DG, DE, and DH; designation of the samples is described in Chapter 3.2. No statistically significant differences (*p* < 0.05) were found in the paired comparative test observing the pleasantness of emulsion matrices with both 5% and 10% flower and forest honey content. Furthermore, the results of a paired test comparing the spreadability of the formulations with 1% and 3% beeswax content were also statistically significant, in favor of a sample with a lower beeswax content. Additions of beeswax can modify the texture and viscosity of the matrices, but also act as a smoothing and opacifying agent [19].

### 2.3. Questionnairre Survey

The questionnaire survey revealed that honey was used by 92% of the respondents, propolis was used by 40% of the respondents, royal jelly was used by 14% of the respondents, wax was used by 35% of the respondents, and bee venom was used by 3% of the respondents. As a reason for using honey cosmetics, 15% interviewed accepted the advice of a friend, 5% decided on the basis of information from the media, and 3% of them stated physician recommendations. As a result of skin problems, 5% searched for bee products, and 61% of those interviewed stated other reasons. Most (88%) women were aware of the healing effects of honey and bee products; meanwhile, 9% of the respondents suffered from allergy to bee products, of which 5% were to bee venom, 3% were to pollen, and 1% were to honey. The processing of honey and bee products was carried out by 4% of respondents. More than half of the respondents had experience with cosmetics containing honey and bee products. Cosmetics with honey and bee products were used by 55% of women. The reason was that these cosmetic products are used to improve psoriasis and dry skin condition (45%), improve the wound state (25%), strengthen immunity (12%), reduce acne (6%), reduce inflammation (6%), reduce eczema (3%), and relieve pain (3%). The cosmetic products were most often applied to the body, face, hands, eye area, hair, and oral cavity. The most common forms of cosmetic products were toothpastes, creams, ointments, balms, tinctures, lotions, masks, mouthwashes, gels, and shampoos.

## 3. Materials and Methods

### 3.1. Analysis of Forest and Flower Honey

The analysis of forest and flower honey was carried out at the Institute of Environment of the Faculty of Technology (Zlín, Czech Republic) in cooperation with the Slovak Academy of Sciences (Bratislava, Slovak Republic) see Table 1. Moisture was determined by refractometric method according to DIN 10752 [31]. The principle is that the water content is determined from the refractive index of honey (which increases with the dry matter content). The acidity was determined by titrating a sample of honey dissolved in 0.1 mol/L NaOH according to Lord et al.; acidity expresses the amount of all free acids in honey [32]. The mineral content was determined gravimetrically after burning and annealing the sample at 600 °C [33]. Amino acids were determined by gas chromatography with flame ionization and mass spectrometric detection (Shimadzu, Tokyo, Japan) [34,35]. The content of reducing saccharides (glucose, fructose, maltose), non-reducing disaccharide (sucrose) and oligosaccharides was determined by high-performance liquid chromatography with a refractive index detector (Shimadzu, Tokyo, Japan) [36,37,38]. Water-soluble vitamins (B2, B3, B5, B9, and C) were determined by reversed phase high-performance liquid chromatography [39]. Enzyme (diastase) was determined according to methods described by Edwards et al. [40] and Hadorn [41].

### 3.2. Procedure of Preparation of Cosmetics Matrices

A total of 12 emulsion matrices containing *Apis mellifera* European honey and bee products were prepared in various concentrations, selected according to available sources [7,12,42,43,44] and specifications from manufacturers [45,46]. The aim of the study was to test the effectiveness of recommended minimal and maximal additions of honey and bee products into an emulsion base (EB). To prepare the matrices, the EB (Fagron, Czech Republic) with following composition (according to International Nomenclature of Cosmetic Ingredients) was used: *Aqua, Paraffin, Paraffinum Liquidum, Cetearyl Alcohol, Laureth 4, Sodium Hydroxide, Carbomer, Methylparaben, Propylpareben*. Part of the honey whose skin effect was studied came from colonies located on the roof of the Faculty of Technology. The list of formulations is as follows. EB with the addition of flower honey (Tomas Bata University in Zlín, Czech Republic) in concentrations of 5% and 10% (samples A, B) and forest honey (Hostyn-Vsetin Highlands, 25 km far from Tomas Bata University in Zlín, Czech Republic) in concentrations of 5% and 10% (samples C, D). Other products added to the basic formulation were glycerin–aqua–mel extract PHYTAMI^®^ HONEY-F06 (Alban Mueller International, France) at concentrations of 2% and 10% (samples E, F), aqua–mel extract CRODAROM HONEY (Crodarom, France) at concentrations of 2% and 10% (samples G, H), beeswax (Včelpo, Czech Republic) at concentrations of 1% and 3% (samples I, J), royal jelly (Včelpo, Czech Republic) at a concentration of 0.5% (sample K), and propolis (Včelpo, Czech Republic) at a concentration of 1% (sample L). 

The procedure for preparing the matrices was chosen with respect to the particular honey or bee products. Flower honey, forest honey, and royal jelly were homogenized into EB on a Heidolph stirrer at 2000 rpm for 10 min at room temperature. Propolis and beeswax had to be treated before mixing into EB. An ethanolic tincture was formed from the crude propolis by mixing 1 part propolis (100 mL) and 2 parts of 96% ethanol (200 mL). This mixture was left to infuse with occasional shaking for 7 days and then filtered and added to EB. Beeswax creams were prepared by weighing the required amount of wax and EB separately. The wax was heated in a water bath at 70 °C for 10 min, and the heated wax was added to EB at 60 °C. This was followed by homogenization with a Heidolph stirrer at 2000 rpm at laboratory temperature.

### 3.3. Instrumental Techniques and Study Design

In the field of experimental dermatology and cosmetology, non-invasive methods are widely used, which allow quantitative evaluation of parameters describing the barrier function of the skin. These methods were also applied in this study. The CORNEOMETR^®^ CM 825 corneometric probe (Courage & Kazaka Electronic, Cologne, Germany) was used to measure the water content of the *stratum corneum*, based on the evaluation of changes in electrical capacity on the skin surface, using relatively high dielectric water constants. The results are displayed in arbitrary units (a.u.). Another parameter measured was transepidermal water loss (TEWL), which was monitored by a TEWAMETER^®^ TM 300 probe (Courage & Kazaka Electronic, Cologne, Germany). In principle, the flow of water vapor above the *stratum corneum* into the open chamber of a cylindrical shape with two pairs of sensors for temperature and relative humidity is determined. TEWL is calculated from the difference between the two measurement points using Fick’s law of diffusion and displayed in grams per square meter per hour (g/m^2^/h). Skin-pH-meter^®^ PH 905 (Courage & Kazaka Electronic, Cologne, Germany) was used to determine skin acidity. The specially designed probe consists of a flat-topped glass electrode for full skin contact, which was connected to a voltmeter. The system measures potential changes due to the activity of hydrogen cations surrounding the very thin layer of semisolid forms at the top of the probe. The changes in voltage are displayed as pH.

Measurements were performed on 24 healthy women (aged 23 to 49 years, mean age 36 years) with no history of atopic eczema or other skin diseases. The volunteers were divided into two groups, each testing six formulations (see the study design in Table 2). Volunteers were acquainted in advance with the purpose and course of the measurement. Informed consent was obtained from all of them, and said study was approved by the International Ethical Guidelines for Health-Related Research Involving Humans [47]. For 12 h prior to and during the study, the volunteers were not allowed to apply any topical cosmetic products; only an evening shower water was permitted. Measurements were carried out in an air-conditioned room (temperature 22.0−24.0 °C, relative humidity 45.0−50.0%). All measurements were performed after a rest of 20 min for equilibration. The volar side of the forearm of the right and left hand was divided into test sites with an area of 8 cm^2^ (see design in Table 2), which were pretreated for 4 h with 0.5% SLS solution (Sigma-Aldrich, Czech Republic) prepared in saline. After this pretreatment, the following indicators were measured on each test site: hydration with a corneometric probe, TEWL using a tewameter, and acidity of the skin by a pH probe. The untreated spot, designated as a control, was used to compare any irritant reactions to the skin. Then, 0.5% SLS solution and honey and bee products matrices were applied to each site. The effect of the applied samples on the *stratum corneum* was monitored in all volunteers after 1, 2, 3, 4, 24, and 48 h in the same order as after SLS treatment. Hydration was measured five times at each test site. TEWL measurements were performed 15 times at each test site. Since this is dependent on skin temperature, ambient temperature, and the temperature of a probe itself, the first five values were eliminated.

### 3.4. Sensory Analysis and Questionnairre Survey

The panel of assessors consisted of a total of 12 assessors at a trained assessor level. The assessors were acquainted with the objective of analysis and instructed on the way of evaluation of samples of individual products. The sensory evaluation and equipment of the sensory laboratory were in compliance with regulations defined by International Standards ISO 6658 [48] and ISO 8589 [49]. The temperature in the room was at 20.0–22.0 °C, relative humidity 45.0–50.0% under conditions of artificial light. The sensory analysis included ordinal tests focused on overall sample preference and sample color preference for 8 selected formulations: containing 5% and 10% flower honey (samples A and B), 5% and 10% forest honey (samples C and D), 2% and 10% glycerin–aqua–mel extract (samples E and F), 2% and 10% aqua–mel extract (samples G and H). Then, paired comparative tests for honey and beeswax formulations were included. The first paired test examined the comfort of the 5% flower and forest honey samples on the skin. Another paired test evaluated a pair of samples with 10% content of flower and forest honey in the same characteristics. The last paired test evaluated the spreadability of cream samples containing 1% and 3% beeswax.

A questionnaire survey was proposed to ascertain data for the use of honey and bee products in cosmetics and to map the existence of knowledge about the effects of these products. The questionnaire included socio-demographic questions determining age (15–20 years, 21–30 years, 31–40 years, 41–50 years, 51–60 years, 61–70 years), place of residence (village, town, city), education (elementary, apprenticeship, apprenticeship with school-leaving exam, secondary, higher professional and university education) and field of the employment of respondents: health service, education, food industry, services (cosmetic, wellness, hairdressing), nutrition consultancy, state administration, and others. This was followed by questions focused on honey and bee products, which are presented in Table 3. The survey was anonymous, the target group of respondents were women. In total, 120 questionnaires were distributed with a return of 83%. The proportion of respondents in individual age categories was even except for respondents aged 61 to 70, which was only 5%. Most respondents had secondary and university education (43% and 51%).

### 3.5. Statistical Analysis

Analyses of forest and flower honey were performed in triplicate; the arithmetic mean and standard deviation values were calculated using Microsoft Office Excel 2013 (Microsoft, Santa Rosa, California, CA, USA). Hydration, TEWL, and pH values were recorded and processed via MPA 5 station operating software (Courage & Khazaka Electronic, Cologne, Germany). The results of biophysical characteristics reported as the mean values with standard deviations were carried out in Excell software (version 10, Microsoft, Santa Rosa, California, CA, USA). The results of sensory analysis—ordinal and pair tests—were evaluated by Friedman’s test at 5% significance level. Frequency expression was used to evaluate the data obtained by the questionnaire survey.

## 4. Conclusions

Between selected honey and bee products, there are differences in the ability to hydrate the skin and improve its barrier properties, including adjusting the acidity of the skin surface. Their effectiveness is dependent on the type and concentration of the product incorporated into the cosmetic vehicle. Very good moisturizing properties have been found in emulsion matrices with a glycerin extract of honey, which is attributed to the synergistic effect of glycerin present, which is a traditional humectant very often used in cosmetic products. Cosmetic matricess containing higher concentrations of honey or bee products hydrated the skin more effectively except for the aqua–mel extract formulation where lower concentrations were found to be more favorable. A very interesting difference in the ability to hydrate was observed in forest and flower honey formulations, where forest honey had a higher hydrating activity. Even the skin treated with matrices containing other bee products had a higher proportion of water in its corner layer. Barrier properties were enhanced by gradual absorption, especially in samples with both glycerin and aqueous honey extract. A favorable finding from the measurement results was the shift of the skin pH to the neutral area. By sensory analysis, differences in color of emulsion matrices were evaluated from organoleptic properties. A paired test found a difference in the spreadability of formulations with different amounts of beeswax. The reasons and frequency of using of cosmetics containing honey and bee products vary. Summarizing the information obtained, honey and bee products as substances of natural origin are very popular and traditionally used primarily for their unique effects not only in cosmetics, but also in many other areas. Cosmetic matrices enriched with honey or bee products are suitable for the care of skin which is repeatedly exposed to surfactants contained in cosmetic personal care products and in variety of cleansing agents.

## Figures and Tables

**Table 1:** Composition of forest and flower honey.

Component	Forest Honey (% ± SD)	Flower Honey (% ± SD)
Fructose	31.30 ± 1.95	38.40 ± 2.01
Glucose	36.80 ± 4.60	33.20 ± 4.08
Sucrose	2.30 ± 0.59	2.90 ± 0.55
Maltose	5.50 ± 0.48	3.10 ± 0.34
Oligosaccharides	3.70 ± 0.61	2.20 ± 0.41
Moisture	18.60 ± 2.17	18.20 ± 2.04
Enzymes	0.16 ± 0.02	0.56 ± 0.03
Vitamins	0.11 ± 0.02	0.21 ± 0.02
Ash	1.04 ± 0.04	0.45 ± 0.03
Free acids	0.15 ± 0.02	0.35 ± 0.03
Amino acids	0.34 ± 0.02	0.43 ± 0.02

**Table 2:** Design of the volar side of forearms of two groups of volunteers with tested formulations; concentrations of honey, honey extracts, and other bee products are in EB (*w*/*w*).

Volar Side of the Left Forearm		Volar Side of the Right Forearm	
1st group of volunteers	2nd group of volunteers	1st group of volunteers	2nd group of volunteers
Control		Aqua-mel extract 2%	Floral honey 10%
SLS		Aqua-mel extract 10%	Glycerin–aqua–mel extract 2%
Forest honey 5%	Royal jelly 0.5%	Beeswax 1%	Glycerin–aqua–mel extract 10%
Forest honey 10%	Floral honey 5%	Beeswax 3%	Propolis 1%

**Table 3:** Summary of questions and answers possibilities in the questionnaire.

No.	Question
1	Which products do you use? Answer possibility: honey, propolis, royal jelly, wax, bee venom, pollen
2	What reasons led you to use honey and bee products? Answer possibility: physician recommendation, friend’s recommendation, advertising, skin problems, others
3	Do you think bee products has healing effects? Answer possibility: yes/no/don’t know
4	Are you allergic to any of the listed products: honey, propolis, royal jelly, wax, bee venom, pollen? Answer possibility: yes/no
5	Do you process honey or bee products for the production of ointments, tinctures, emulsions, etc. for home use? Answer possibility: yes/no
6	Do you use honey cosmetics? Answer possibility:/no
(a)	What is the reason for using honey cosmetics? Answer possibility: pain relief, wound healing, acne, inflammation, eczema, psoriasis, dry skin, strengthening immunity
(b)	What types of honey cosmetics do you use? Answer possibility: healing cosmetics (drops/tincture/spray/gel/ointment/balm/cream), cleaning cosmetics (wipes/water/milk/peeling/mask/toothpaste/mouthwash), cosmetics for men (shampoo/after shave/men’s cream), body cosmetics (milk/balm/bath foam/soap/shower shampoo), hair cosmetic (shampoo/hair lotion/balm)
